# Bronchial bacterial colonization and the susceptibility of isolated bacteria in patients with lung malignancy

**DOI:** 10.2478/raon-2025-0018

**Published:** 2025-02-27

**Authors:** Sabrina Petrovic, Bojana Beovic, Viktorija Tomic, Marko Bitenc, Mateja Marc Malovrh, Vladimir Dimitric, Dane Luznik, Martina Miklavcic, Tamara Bozic, Tina Gabrovec, Aleksander Sadikov, Ales Rozman

**Affiliations:** 1 Surgery Bitenc, Medical Centre Ljubljana (MCL), Ljubljana, Slovenia; 2 Clinic for Infectious Diseases and Fever Conditions, University Medical Centre Ljubljana, Ljubljana, Slovenia; 3 Faculty of Medicine, University of Ljubljana, Ljubljana, Slovenia; 4 University Clinic of Pulmonary and Allergic Diseases Golnik, Golnik, Slovenia; 4 Faculty of Medicine, University of Ljubljana, Ljubljana, Slovenia; 5 Faculty of Computer and Information Science, University of Ljubljana, Ljubljana, Slovenia

**Keywords:** bronchial bacterial colonization, potentially pathogenic microorganisms, antibiotic prophylaxis, lung cancer, bronchoscopy

## Abstract

**Background:**

Postoperative pneumonia (POP) remains a leading cause of mortality following lung surgery. Recent studies have confirmed that the respiratory tract below the vocal cords is not sterile and often harbours potentially pathogenic microorganisms (PPMs), putting patients with lung malignancies at an increased risk for pulmonary infections.

**Patients and methods:**

The study analysed 149 patients who underwent bronchoscopy for lung lesions suspected to be lung cancer. Protected specimen brush (PSB) samples were obtained during bronchoscopy prior to any specific treatment. Bacterial identification and antimicrobial susceptibility testing were conducted on the isolated strains.

**Results:**

Bacterial colonization was detected in 88.6% of patients, with 21.5% carrying PPMs. Notably, patients with type 2 diabetes exhibited a higher rate of PPM colonization compared to others. Antibiotic susceptibility testing showed no significant differences in efficacy between amoxicillin with clavulanic acid and first-generation cephalosporin in both colonized patients and those with PPMs. Importantly, no multidrug-resistant bacteria were identified.

**Conclusions:**

Our findings indicate a slightly lower PPM colonization rate compared to previous studies, possibly due to the unique geographic characteristics of the study population. The absence of significant differences in bacterial susceptibility between the two tested antibiotics highlights the need for further research to refine perioperative infection management strategies.

## Introduction

Postoperative pneumonia (POP) remains a significant contributor to postoperative mortality following lung surgery, with reported incidence rates ranging from 2% to 20%.^1,2^ Patients with lung malignancies are particularly susceptible to pulmonary infections due to factors such as immunosuppression, impaired protective mechanisms, and localized inflammation caused by concurrent conditions like bronchiectasis and chronic obstructive pulmonary disease (COPD).^2^

Recent studies have challenged the traditional belief that the respiratory tract below the vocal cords is sterile, highlighting the presence of microbial colonization.^3^ However, limited research has focused on bronchial bacterial colonization (BBC) patterns in patients with lung malignancies. Existing studies report a wide range of BBC prevalence, from 10% to 83%, often involving potentially pathogenic microorganisms (PPMs) such as *Haemophilus influenzae, Streptococcus pneumoniae,%* and *Staphylococcus aureus*.^1,2,4,5^ While the clinical significance of these microorganisms within the airways remains uncertain, their presence may influence the management and prognosis of lung cancer patients.^3^ Several risk factors, including age, gender, COPD, and smoking, have been associated with an increased likelihood of PPM colonization.^1,2,4^ Furthermore, studies have established a link between BBC and pneumonia in these patients, though it remains unclear whether these bacteria contribute to postoperative infections after lung surgery.^1^ Nevertheless, PPM colonization of the respiratory tract could elevate the risk of postoperative infections.^2^

The effectiveness of first-generation cephalosporins as perioperative antibiotic prophylaxis, as recommended by current guidelines, is under scrutiny due to the high incidence of postoperative pneumonia and the increasing prevalence of antibiotic-resistant bacteria among isolated strains.^6–9^ Addressing postoperative infections in patients with lung malignancies undergoing surgery is a critical clinical challenge, necessitating the identification of effective prophylactic strategies.

This study aims to prospectively evaluate the prevalence of PPM colonization in patients with lung malignancies, predominantly primary lung cancer, at the time of diagnosis before any specific treatment initiation. Additionally, it investigates antibiotic susceptibility among isolated bacteria to assess resistance rates and examines the potential association between PPM colonization and cancer stage.

## Patients and methods

This prospective study was conducted from June 2021 to February 2023, focusing on patients presenting with lung lesions suspected to be primary lung cancer. During the initial outpatient evaluation, demographic and clinical data were collected, including age, gender, smoking history, and comorbidities. All patients were diagnosed following established guidelines for primary lung cancer diagnosis. TNM staging included chest, abdominal, and head CT scans, as well as PET-CT imaging. Flexible bronchoscopy was performed for all patients to obtain tumour tissue samples for histological diagnosis when possible. In addition, protected specimen brush (PSB) samples were collected during bronchoscopy prior to initiating any specific treatment. For cases where bronchoscopic tumour r access was not feasible, CT-guided needle biopsies were used to determine histological typing.

PSB samples were sent to the microbiology laboratory, where bacterial colonization was defined as the isolation of microorganisms at a threshold of ≥10^3^ CFU/mL. Antimicrobial susceptibility testing was performed on each bacterial isolate using the microbiology protocol tailored to the bacterial species.

The study received approval from the National Medical Ethics Committee of the Republic of Slovenia (no. 0120-163/2021/3), and all participants provided written informed consent.

### Bronchoscopy

Bronchoscopy was performed under moderate sedation, adhering to a strict no-suction policy prior to reaching the carina. Upon entering the trachea, topical lidocaine anaesthesia was administered to the main and upper lobar bronchi. Sterile brushes (OLYMPUS disposable cytology brush BC-202D-210) were used to collect samples from the bronchi of the tumour-bearing lobe prior to diagnostic sampling to detect bacterial colonization. Each sample was preserved in 1 mL of sterile saline solution and sent to the microbiology laboratory. Peripheral tumour sampling was conducted using various bronchoscopic techniques to determine tumour histological types.

### Microbiological analysis

PSB samples were promptly processed in the microbiology laboratory. Samples were vortexed, and slides were prepared before dilution and plating. Gram staining and microscopic examination assessed sample quality, bacterial morphology, and abundance. Samples were diluted to a final concentration of 10^−3^ and inoculated on various solid and liquid media, including blood agar, chocolate agar, Brucella blood agar, CHROMagar™ Orientation (CHROMagar, France), and thioglycollate broth. Plates were incubated aerobically and anaerobically at 35°C and evaluated for growth at 24, 48, and 72 hours. Liquid medium subculturing onto the same solid media plates confirmed bacterial morphotypes and colony-forming units per millilitre (CFU/mL). A threshold of ≥10^3^ CFU/mL was used to define positive culture results.

Bacterial identification and antimicrobial susceptibility testing were performed using the MALDI Biotyper® (Bruker Daltonics GmbH & Co, Germany) and the standardized EUCAST disc diffusion method. Bacteria were classified as PPMs (e.g., S. *pneumoniae, H. influenzae, M. catarrhalis, S. aureus, P. aeruginosa, Enterobacterales*) or non-PPMs (e.g., *Streptococcus viridans group, Neisseria spp., Corynebacterium spp*., coagulase-negative staphylococci).^5^

### Statistical analyses

Descriptive statistics were presented as median (range) for continuous variables and as frequencies and proportions for categorical variables. Comparisons of bacterial colonization rates with respect to tumour stage and comorbidities, as well as antibiotic susceptibility, were assessed using Pearson’s chi-squared test or Fisher’s exact test, as appropriate. A p-value < 0.05 was considered statistically significant. All p-values are two-tailed. Statistical analyses were conducted using IBM SPSS (version 21, Chicago, IL, USA).

## Results

The study included 149 consecutive patients with lung malignancies, with a median age of 66 years (20–84). Baseline characteristics of the participants are summarized in Table 1. Most patients (71.8%) were diagnosed with non-small cell lung cancer, primarily adenocarcinoma (57%).

**TABLE 1.** Baseline characteristics of patients

Characteristics	n	%
Patients	149	
Male	90	60.4
Median age (years)	66	
Smokers	50	33.6
Ex-smokers	71	47,7
Non-smokers	28	18,8
COPD	44	29.5
Diabetes type 2	13	8.7
Colonized patients	132	88.6
Colonized with PPMs	32	21.5
Multiple bacteria colonization	86	57.7
Adenocarcinoma	86	57.7
Squamous cell carcinoma	22	14.8
Small cell carcinoma, carcinoid or large cell carcinoma	11	7.4
Non-small cell carcinoma NOS*	17	11.4
Other, non-lung cancer malignancies (limfoma, methastases)	13	8,7

1 COPD = chronic obstructive pulmonary disease; NOS = not otherwise specified; PPMs = potentially pathogenic microorganisms

Respiratory tract colonization with at least one bacterial strain was confirmed in 132 patients (88.6%), with 86 patients (57.7%) harbouring multiple bacterial strains. Colonization with potentially pathogenic microorganisms (PPMs) was identified in 32 patients (21.5%). Antibiotic sensitivity testing for amoxicillin with clavulanic acid and first-generation cephalosporins was performed in 120 patients. Sensitivity testing for amoxicillin with clavulanic acid and first-generation cephalosporins was not conducted for 12 patients due to colonization with bacteria requiring specific antibiotic panels (*Rothia mucilaginosa, Streptococcus constellatus, Actinomyces odontolyticus, Streptococcus cristatus*, and *Fusobacterium periodonticum*), none of which were classified as PPMs.

The most frequently isolated PPMs were *Haemophilus influenzae, Streptococcus pneumoniae, Staphylococcus aureus*, and *Escherichia coli* (Table 2), while the most common non-PPMs included *Streptococcus mitis* and *Streptococcus salivarius*. Notably, 57.7% of patients exhibited colonization by multiple bacterial strains.

**TABLE 2.** Number and percentage of recovered bacteria

RECOVERED BACTERIA	No. of patients with isolated species	% of patients with isolated species
Streptococcus mitis	53	35,6%
Streptococcus salivarius	36	24,2%
Streptococcus oralis	27	18,1%
Streptococcus parasanguinis	23	15,4%
Streptococcus vestibularis	18	12,1%
Veillonella atypica	13	8,7%
**Haemophilus influenzae**	12	8,1%
**Streptococcus pneumoniae**	11	7,4%
Neisseria subflava	9	6,0%
Actinomyces odontolyticus	9	6,0%
**Staphylococcus aureus**	8	5,4%
Haemophilus parahaemolyticus	8	5,4%
Streptococcus gordonii	8	5,4%
Rothia mucilaginosa	7	4,7%
**Escherichia coli**	6	4,0%
Staphylococcus epidermidis	6	4,0%
Staphylococcus hominis	4	2,7%
Streptococcus anginosus	4	2,7%
Veillonella parvula	3	2,0%
Fusobacterium periodonticum	3	2,0%
**Moraxella catarrhalis**	2	1,3%
**Pseudomonas aeruginosa**	2	1,3%
**Haemophilus parainfluenzae**	2	1,3%
Corynebacterium simulans	2	1,3%
Prevotella nigrescens	2	1,3%
Streptococcus constellatus	2	1,3%
Gemella haemolysans	2	1,3%
**Serratia marcescens**	2	1,3%
Prevotella melaninogenica	2	1,3%
Granulicatella adiacens	2	1,3%
Streptococcus agalactiae	1	0,7%
Staphylococcus capitis	1	0,7%
Streptococcus cristatus	1	0,7%
Neisseria macacae	1	0,7%
Neisseria cinerea	1	0,7%
Neisseria flavescens	1	0,7%
Veillonella dispar	1	0,7%
Prevotella jejuni	1	0,7%
Campylobacter concisus	1	0,7%
**Citrobacter koseri**	1	0,7%
Prevotella pallens	1	0,7%
**Enterobacter bugandensis**	1	0,7%
**Acinetobacter lwoffii**	1	0,7%
Moraxella nonliquefaciens	1	0,7%

No statistically significant differences in PPM colonization rates were observed across different cancer stages (Table 3). Similarly, no significant association was found between COPD and colonization with potentially pathogenic bacteria (p = 0.39) (Table 4). However, type 2 diabetes emerged as an independent risk factor for colonization with potentially pathogenic bacteria (p = 0.04) (Table 5).

**TABLE 3.** Relationship between cancer stage and colonization with potentially pathogenic microorganisms (PPMs)

STAGE (8th TNM classification)	PPMs		Total
	no	yes	
**I**	50	11	61
	82.0%	18.0%	100.0%
**II**	25	7	32
	78.1%	21.9%	100.0%
**III**	16	6	22
	72.7%	27.3%	100.0%
**IV**	11	2	13
	84.6%	15.4%	100.0%
**Total**	102	26	128*
	79.7%	20.3%	100.0%

* for patients, who didn’t have primary lung cancer, cTNM was not defined

**TABLE 4.** Relationship between colonization with potentially pathogeni microorganisms (PPMs) and chronic obstructive pulmonary disease (COPD)

COPD	PPMs		Total
	no	yes	
**no**	83	21	104
	79.8%	20.2%	100.0%
**yes**	32	12	44
	72.7%	27.3%	100.0%
**Total**	115	33	148*
	77.7%	22.3%	100.0%

* for 1 patient, there was no comorbidity data

**TABLE 5.** Relationship between colonization with potentially pathogenic microorganisms (PPMs) and diabetes type 2

DIABETES TYPE 2	PPMs		Total
	no	yes	
**no**	108	27	135
	80.0%	20.0%	100.0%
**yes**	7	6	13
	53.8%	46.2%	100.0%
**Total**	115	33	148*
	77.7%	22.3%	100.0%

* for 1 patient, there was no comorbidity data

Antibiotic susceptibility testing revealed no significant differences in efficacy between amoxicillin with clavulanic acid and first-generation cephalosporin in both colonized patients and those colonized specifically by PPMs (Tables 6 and 7).

**TABLE 6.** Susceptibility among all colonized patients

Amoxicillin with clavulanic acid	First generation cephalosporin			Total
	R	S	S/R	
**R**	2	2	0	4
**S**	2	101	1	104
**S/R**	0	6	6	12
**Total**	4	109	7	120

1 R = resistant; S = susceptible

**TABLE 7.** Susceptibility among patients colonized by potentially pathogenic microorganisms (PPMs)

Amoxicillin with clavulanic acid	First generation cephalosporin			Total
	R	S	S/R	
**R**	1	1	0	2
**S**	1	20	0	21
**S/R**	0	5	3	8
**Total**	2	26	3	31

1 R = resistant; S = susceptible

## Discussion

In this study, we conducted a prospective investigation of BBC in patients suspected of primary lung cancer before initiating any treatment. Our methodology introduced a key distinction from previous studies by using sterile brush specimens to collect samples from the bronchi of the tumourcontaining lobe. Additionally, we evaluated the antibiotic susceptibility of isolated bacteria to antibiotics commonly used for perioperative prophylaxis in thoracic surgery.

Our findings revealed a lower prevalence of colonization by PPMs (21.5%) compared to previous studies. Only two patients harboured bacteria resistant to both amoxicillin with clavulanic acid and first-generation cephalosporin. In one instance, bacteria were resistant to amoxicillin with clavulanic acid but susceptible to first-generation cephalosporin, while the reverse was observed in another case. Importantly, there were no significant differences in susceptibility between the two antibiotics, and no multidrug-resistant bacteria were identified.

In a similar study, Laroumagne *et al*. examined bronchial colonization at the time of lung cancer diagnosis. They reported a higher prevalence of PPM colonization (50%), likely due to non-sterile sampling conditions. Their findings suggested an association between bronchial colonization and lower survival rates, potentially linked to infectious complications.^4^

Ioanas *et al*. reported a PPM colonization rate of 41%, again using non-sterile sampling techniques. Their study demonstrated no resistance to conventional antibiotics, consistent with our findings. They also reported a low incidence of postoperative pulmonary infections (12%) and no pneumonia cases, likely attributable to effective prophylaxis with first-generation cephalosporin administered perioperatively and for 48 hours postoperatively. Similar complication rates were observed in colonized and non-colonized patients, although their study was limited to 41 patients.^5^

Dancewicz *et al*. also reported similar BBC rates and found no evidence of multidrug-resistant microorganisms, aligning with our results.^2^ Boldt *et al*., however, reported a PPM colonization rate of 48% in patients undergoing lung surgery. They found that a single dose of sulbactam plus ampicillin was significantly more effective than firstgeneration cephalosporin in preventing infections, suggesting alternative regimens for prophylaxis.^10^

Radu *et al*. conducted a retrospective analysis of 312 cases, highlighting the inefficacy of firstgeneration cephalosporin in 84% of cases, raising concerns about current prophylactic guidelines.^8^ Schlusser *et al*. suggested that targeted antibiotic prophylaxis against bronchial colonizing bacteria could reduce postoperative pneumonia incidence. They observed a significant reduction when antibiotics were tailored to the identified bacteria, though their study was not randomized and warrants further validation.^6,7^

Lastly, D′Journo*et al.′s* meta-analysis established a statistical correlation between preoperative BBC and postoperative respiratory complications, emphasizing the clinical importance of preoperative colonization screening.^1^

## Conclusions

This study provides valuable insights into bronchial bacterial colonization in patients with lung malignancies, predominantly primary lung cancer. The prevalence of PPM colonization and the low resistance to tested antibiotics characterize a patient population primarily from central and western Slovenia, differing from studies conducted in other geographical regions. While PPM colonization was not associated with lung cancer stage or COPD, a significantly higher prevalence was observed in patients with type 2 diabetes.

The absence of significant differences in antibiotic susceptibility between amoxicillin with clavulanic acid and first-generation cephalosporin highlights the need for further research. Given the substantial rates of colonization and postoperative pneumonia, we recommend routine microbiological sampling during bronchoscopy for all patients suspected of primary lung cancer. This approach could enable targeted perioperative antibiotic prophylaxis in patients undergoing thoracic surgery. Future prospective studies comparing targeted *versus* standard prophylaxis are essential to establish best practices.
